# Similarities and differences in gene expression profiles of *BRCA1* methylated and mutated epithelial ovarian cancers

**DOI:** 10.3389/fonc.2023.1268127

**Published:** 2023-10-03

**Authors:** Nora Sahnane, Laura Libera, Sofia Facchi, Ileana Carnevali, Susanna Ronchi, Chiara Albeni, Antonella Cromi, Jvan Casarin, Fausto Sessa, Maria Grazia Tibiletti

**Affiliations:** ^1^ Unit of Pathology, Azienda Socio Sanitaria Territoriale (ASST) Sette Laghi, Varese, Italy; ^2^ Research Centre for the Study of Hereditary and Familial Tumors, University of Insubria, Varese, Italy; ^3^ Department of Medicine and Technological Innovation, University of Insubria, Varese, Italy; ^4^ Obstetrics and Gynaecology Department, Del Ponte Women’s and Children’s Hospital, Varese, Italy

**Keywords:** *BRCA1* methylation, NanoString^®^, gene expression profiles, PARPi therapy, EOC

## Abstract

**Introduction:**

*BRCA1* methylated (*BRCA1^met^*) epithelial ovarian cancer (EOC) is a recently defined and not well-investigated subset of neoplasms. To date, no studies have focused on the transcriptional profiles of *BRCA1^met^* cases, and, as a matter of fact, we still do not know if this subset of EOCs is similar, and to what extent, to *BRCA1* mutated (*BRCA1^mut^*) cases.

**Methods:**

We compared a group of 17 *BRCA1^met^* cases against 10 *BRCA1^mut^* cases using a subset of carefully selected 17 *BRCA^wt^* EOCs as a control group.

**Results:**

First, *BRCA1^met^* cases showed a downregulation of the relative transcript, while this association was not observed for *BRCA1^mut^* EOCs. The *BRCA1^met^* group exhibited a general upregulation of homologous recombination (HR)-related genes, as well as *BRCA1^mut^*. Overall, *BRCA1^met^* had a different gene expression profile, characterized by diffuse downregulation, whereas *BRCA1^mut^* showed a general upregulation (p < 0.0001). Both *BRCA1*-defective groups showed a slightly activated immune response mediated by interferon (IFN) gamma pathways.

**Discussion:**

In conclusion, even if the expression profile of many genes related to DNA damage and repair system is shared between *BRCA1^mut^* and *BRCA1^met^* EOCs supporting that *BRCA1^met^* EOCs may benefit from PARPi therapies, our data demonstrate that *BRCA1^mut^* and *BRCA1^met^* EOCs show different expression profiles, suggesting a different mechanism of carcinogenesis that can be reflected in different responses to therapies and disease recovery.

## 1 Introduction

Epithelial ovarian cancer (EOC) is the most common lethal malignancy among gynecological cancers with a poor outcome due to late diagnosis caused by a lack of early signs and symptoms of this disease (1). The standard treatment for EOCs is cytoreductive surgery and platinum-based chemotherapy. Recently, therapy with PARP inhibitors (PARPi) has been proven to be effective for patients with EOC showing homologous recombination deficiency (HRD), which is mainly observed in EOCs harboring germinal/somatic pathogenetic variants or promoter methylation of the genes involved in homologous recombination (HR) pathway, mainly *BRCA1* and *BRCA2* genes (2, 3). In Italy, PARPi therapy has been approved as a maintenance treatment for patients with HRD EOCs in the front-line setting after chemotherapy as well as in the second-line treatment of recurrent platinum-sensitive disease regardless of HRD status. However, the most important predictive biomarkers for PARPi sensitivity are *BRCA1* and *BRCA2* pathogenetic variants and more recently HRD status (4–7).

In recent times, a growing body of research has identified a subgroup of EOCs that exhibit a phenomenon called promoter hypermethylation of *BRCA* genes. Interestingly, this subgroup is even more prevalent than the subgroup of EOCs with somatic *BRCA* mutations. Moreover, patients with *BRCA*-methylated EOCs tend to have a more favorable prognosis compared to those with unmethylated EOCs (8–11). Despite these findings, the nature of *BRCA*-methylated EOCs remains poorly understood, particularly in the context of personalized cancer therapy. No studies have yet investigated the expression patterns of these cases in relation to EOCs with *BRCA* mutations. It is still uncertain whether *BRCA*-methylated cases share a similar transcriptional profile with mutated cases. Furthermore, from a clinical perspective, it is unclear whether this subset of cases exhibits the same sensitivity to platinum-based and PARPi therapies as cases with *BRCA* mutations do.

To this aim, the expression profiles of 750 genes were analyzed in a series of 47 EOCs composed of methylated *BRCA* (*BRCA^met^*), *BRCA1*-mutated (*BRCA^mut^*), and *BRCA* wild-type/unmethylated (*BRCA^wt^*) EOCs in order to find out the differences and similarities between epigenetically and genetically *BRCA*-defective groups and to explore a biomarker signature, if any, that could better predict response and sensitivity to platinum and PARPi therapies in these subsets of EOCs.

## 2 Materials and methods

### 2.1 EOC series

In this retrospective study, we analyzed a total of 47 samples of formalin-fixed and paraffin-embedded (FFPE) EOCs collected in the Department of Pathology ASST Sette Laghi in Varese (Italy) from 2008 to 2019. All EOCs were tested for somatic and germline *BRCA1/2* variants and *BRCA1/2* promoter methylation.

Three subsets of EOCs were selected: group A (called *BRCA^wt^*), which includes 17 samples from women who tested negative for germline and somatic *BRCA1* and *BRCA2* variants and negative for other genes including HR-related genes (namely, *ATM*, *BRCA1*, *BRCA2*, *BARD1*, *BLM*, *BRIP1*, *CDH1*, *CHEK2*, *EPCAM*, *FAM175A*, *MEN1*, *MLH1*, *MRE11A*, *MSH2*, *MSH6*, *MUTYH*, *NBN*, *PALB2*, *PARP1*, *PARP2*, *PMS2*, *PTEN*, *RAD50*, *RAD51*, *RAD51C*, *RAD51D*, *RBBP8*, *STK11*, *TERF2*, *TOPBP1*, *TP53*, *XRCC1*, *XRCC2*, and *ZNF423*), as reported by Salvati and colleagues (12). In this subset of cases, large rearrangements were not investigated in a somatic setting. Group B (called *BRCA1^mut^*) includes 10 EOCs from women with germline Class 4 or Class 5 variants in *BRCA1* gene. Group C (called *BRCA^met^*), the group of interest, was composed of 20 EOCs that showed somatic methylation of *BRCA1* (n = 16), *BRCA2* (n = 3), or both (n = 1) gene promoters. It is worth mentioning that the *BRCA1* methylation test was conducted using a customized procedure that targeted the promoter regions associated with gene silencing, as previously outlined in the study conducted by Sahnane et al. (9). For the *BRCA1* test, we performed a cross-validation study with another Italian laboratory to technically validate the assay (8). The *BRCA2* test was designed as described by Vos et al. and Sahnane et al. (9, 13).

All primary EOCs were reviewed and classified by two independent pathology experts in gynecopathology according to the new 2020 World Health Organization (WHO) classification system (14). Ninety-two percent of EOCs were high-grade, and the most frequent histotype was serous (73%). The mean age of onset in the *BRCA^mut^* group was significantly lower than in the *BRCA^met^* group (49.2 versus 58 years, respectively; p = 0.0083; **Supplementary Table 1**).

All analyses were performed in agreement with the Declaration of Helsinki, and the study was approved by the Research Ethics Committee of ATS Insubria (ID 238/2018).

### 2.2 RNA isolation and NanoString nCounter^®^ PanCancer IO360

Total RNA was obtained after manual microdissection from three representative FFPE sections (8 µm) of 47 ovarian tumor samples using the Maxwell^®^ RNA FFPE Kit and Maxwell 16 system (Promega, Madison, WI, USA) according to the recommendations of the manufacturer. RNA was quantified using Qubit™ RNA XR Assay Kit (Invitrogen–Thermo Fisher Scientific, Waltham, MA, USA) and conserved at −80°C until use.

Gene expression analysis was conducted on the NanoString nCounter^®^ gene expression platform (NanoString Technologies, Seattle, WA, USA) using the NanoString PanCancer IO 360™ code set, as we previously described in Bolzacchini et al. (15). NanoString nCounter^®^ PanCancer IO360 code set included 20 reference genes and 750 target genes that were grouped in 13 categories as listed in **Supplementary Table 2**, such as Release of Cancer Cell Antigens (74 genes involved in DNA damage response and repair), Tumor-Intrinsic Factors (155 genes), and Common Signalling Pathways (162 genes included in Wnt, Hedgehog, TGF-beta, NF-kB, Notch, PI3K-Akt, and RAS-MAPK pathways). In detail, a total of 250–300 ng of RNA per sample was mixed with a 3′ biotinylated capture probe and a 5′ reporter probe tagged with a fluorescent barcode. The probes and target transcripts were hybridized overnight at 65°C for 12–16 h, according to the manufacturer’s recommendations. The hybridized samples were run on the NanoString nCounter^®^ preparation station using the high-sensitivity protocol and scanned at high resolution (280 fields of view (FOVs)) on the nCounter Digital Analyzer. The resulting data file in RCC format was used for data analysis.

### 2.3 NanoString nCounter^®^ PanCancer IO360 analysis and statistics

The RCC data for each sample were normalized to internal controls by using nSolver 4.0 software. The obtained counts were then normalized to the geometric mean of 20 endogenous housekeeping genes followed by log2 transformation. Gene expression signatures were calculated as a weighted linear average of the constituent genes (15). The weighted scores used for the calculation of the signatures are NanoString^®^ intellectual property. Normalized gene counts and signature scores were compared to molecular and clinical features. The log2 fold-change, Wald-type confidence interval, and p-value were calculated for each gene and signature.

STRING database of known and predicted interaction was used to identify any possible protein–protein interactions between differentially expressed genes obtained by NanoString^®^ analysis (16). The interactions included direct (physical) and indirect (functional) associations (from computational prediction, knowledge transfer between organisms, and interactions aggregated from primary databases).

Gene list enrichment analysis was performed on April 2023 using ToppGene Suite (17) (updated 8-ago-2022), a one-stop portal for gene list enrichment analysis and candidate gene prioritization, by using the ToppFun tool. The analysis uses 14 annotation categories including gene ontology (GO) terms, pathways, protein–protein interactions, protein functional domains, transcription factor-binding sites, microRNAs, tissue-specific gene expression, and literature. The enrichment significance cutoff level was set to 0.05.

GraphPad v.5.0 (GraphPad Software Inc., San Diego, CA, USA) software was used for the statistical analysis. In detail, Student’s t-test and ANOVA followed by the Bonferroni test, Pearson’s chi-square test, and linear regression analyses were performed, and a p-value of <0.05 was considered significant.

## 3 Results

### 3.1 *BRCA1*-methylated EOC showed downregulation of *BRCA1* transcript

**Figure 1:**
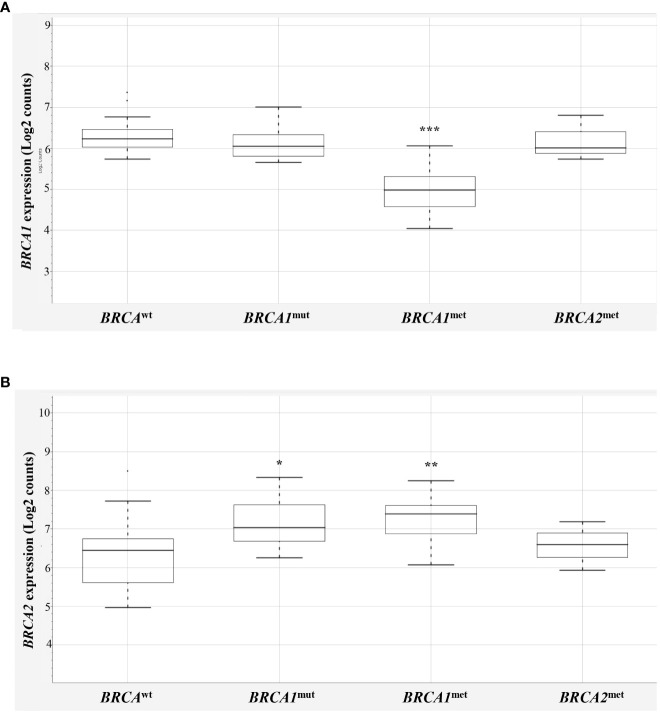
*BRCA1* and *BRCA2* expression in EOCs. Box-plot distribution of *BRCA1* **(A)** and *BRCA2* **(B)** mRNA expression in *BRCA* ^wt^, *BRCA1* ^mut^, *BRCA1* ^met^, and *BRCA2* ^met^ EOCs. The expression data are reported as log2 absolute counts of each gene for each sample. Significant downregulation/upregulation of *BRCA1* or *BRCA2* genes is marked by asterisks. Legend: * p = 0.02–0.05; ** p = 0.0001–0.01; *** p < 0.0001. EOC, epithelial ovarian cancer.

First, to determine if *BRCA1* methylated cases (*BRCA1^met^*) actually showed downregulation of *BRCA1* gene transcript, their *BRCA1* mRNA levels were compared to those observed in *BRCA^wt^* EOCs (**Figure 1A**, **Supplementary Table 1**). A significant downregulation of *BRCA1* transcript was observed for the 17 *BRCA1* methylated cases (one of those showed both *BRCA1* and *BRCA2* methylation) compared to *BRCA^wt^*, with a mean value of log2 counts of 4.94 and 5.9, respectively (p < 0.0001, **Figure 1A**). Highly *BRCA1* methylated (methylation level ≥50%, n = 8) and low *BRCA1* methylated (methylation level at 15%–49%, n = 9) EOCs showed a similar degree of *BRCA1* downregulation (mean log2 counts 5.2 *vs.* 4.7, p-value = 0.1709). Even with a *BRCA1* methylation cutoff of 70%, no differences in BRCA1 transcript levels between high and low methylation cases were observed (respectively, 4.9 *vs.* 5.2, p-value = 0.4925). In the three *BRCA2* methylated cases, no differences in *BRCA1* expression (5.92 log2 counts) were observed in comparison to *BRCA^wt^* EOCs.

Interestingly, *BRCA1* expression evaluation considering the *BRCA1^mut^* group did not highlight differences versus *BRCA^wt^* EOCs, indicating that *BRCA1* mutated cases did not display a downregulation of *BRCA1* expression levels. No differences were recorded considering truncating or stop variants (n = 8) versus missense variants (n = 2, **Supplementary Table 1**), although very few cases were available for this analysis. As reported in the literature (18), the majority of EOCs have a complex chromosome constitution showing several polysomies and of consequence several BRCA1 alleles. On this ground, it is expected that *BRCA1^mut^* transcripts are comparable with *BRCA^wt^* transcripts. In addition, NanoString^®^ probes detecting *BRCA1* and *BRCA2* mRNAs map on exon 13–14 junction of *BRCA1* and on exon 10 of *BRCA2* transcripts.

Then, *BRCA2* expression was compared in the four *BRCA2*-methylated cases (one of those showed both *BRCA1* and *BRCA2* methylation) with respect to the *BRCA^wt^* group, but, unexpectedly, no significant differences between the two groups were observed (6.27 *vs.* 6.03 mean log2 counts, respectively; **Figure 1B**). This finding may suggest that the *BRCA2* methylation test did not address informative *BRCA2* promoter regions—if any—that are associated with gene silencing, casting doubts about the performance of the *BRCA2* methylation test used. Thus, to keep data consistency and exclude any possible bias, the three *BRCA2* methylated cases were eliminated from further analyses (**Supplementary Table 1**, ID# 28, 29, 39).

Surprisingly, *BRCA1*-defective classes (10 *BRCA1^mut^* and 17 BRCA1^met^) showed a significant upregulation of *BRCA2* mRNA versus *BRCA^wt^* class (7.24 and 7.21, respectively versus 6.03 mean log2 counts, p = 0.0122 and p = 0.0039, **Figure 1B**).

### 3.2 DNA damage and repair system genes are upregulated in *BRCA1*-defective EOCs

Based on what was observed for *BRCA2* gene expression and in order to understand if other genes with overlapping functions may compensate for the putative impairment of *BRCA1*, we compared the expression of 61 genes involved in DNA damage and repair in *BRCA*-defective cases with *BRCA^wt^* EOCs (**Supplementary Table 3**, all genes). We observed that, beyond *BRCA1* and *BRCA2* genes, the other 17 genes involved in DNA damage and repair were differently expressed (DE) in *BRCA1*-defective cases with respect to *BRCA^wt^* (**Table 1**, **Supplementary Table 1** and **Supplementary Figure 1**). In detail, in both *BRCA1-*defective groups, *HELLS*, *MRE11*, *UBE2C*, and *UBE2T* were upregulated with respect to *BRCA^wt^* EOC. Nine genes (*BRIP1*, *DTX3L*, *H2AFX*, *MSH2*, *PARP12*, *PARP9*, *RAD51*, *TYMS*, and *WDR76*) were upregulated in *BRCA1^mut^* only, whereas *EXO1*, *FANCA*, and *MGMT* were upregulated exclusively in *BRCA1^met^* cases versus *BRCA^wt^* EOCs. Curiously, in *BRCA1^met^*, we found solely one gene downregulated with respect to *BRCA^wt^* EOCs, namely, the *DDB2* gene. Interestingly, the *DDB2* promoter region contains a response element for p53-BRCA1-E2F downstream of the transcription initiation site, and it has been demonstrated that *BRCA1* downregulation leads to the downregulation of *DDB2* expression (19, 20).

**Table 1:** Significantly upregulated/downregulated DNA damage and/or repair genes in *BRCA*-defective cases (*BRCA1^mut^* and *BRCA1^met^*) compared to *BRCA^wt^* EOCs.

Gene	EOC class	up/down	logFC *BRCA1^mut^*	p-Value *BRCA1^mut^*	logFC *BRCA1^met^*	p-Value *BRCA1^met^*
***BRCA2***	Both	**Up**	0.7104	***0.02507***	0.8071	***0.003725***
***HELLS***	Both	**Up**	0.6212	***0.04503***	0.672	***0.01283***
***MRE11***	Both	**Up**	0.4772	***0.04059***	0.4808	***0.01745***
***UBE2C***	Both	**Up**	1.115	***0.02211***	1.243	***0.00362***
***UBE2T***	Both	**Up**	0.6382	***0.0223***	0.4691	***0.04925***
***BRIP1***	*BRCA1^mut^*	**Up**	0.661	***0.04552***		*ns*
***DTX3L***	*BRCA1^mut^*	**Up**	0.5075	***0.03099***		*ns*
***H2AFX***	*BRCA1^mut^*	**Up**	0.5668	***0.03508***		*ns*
***MSH2***	*BRCA1^mut^*	**Up**	0.6451	***0.01483***		*ns*
***PARP12***	*BRCA1^mut^*	**Up**	0.696	***0.01299***		*ns*
***PARP9***	*BRCA1^mut^*	**Up**	0.5733	***0.02512***		*ns*
***RAD51***	*BRCA1^mut^*	**Up**	0.6283	***0.02263***		*ns*
***TYMS***	*BRCA1^mut^*	**Up**	0.8746	***0.04261***		*ns*
***WDR76***	*BRCA1^mut^*	**Up**	0.9066	***0.004577***		*ns*
***BRCA1***	*BRCA1^met^*	**Down**		*ns*	−1.318	***2.25E−09***
***DDB2***	*BRCA1^met^*	**Down**		*ns*	−0.5902	***0.03501***
***EXO1***	*BRCA1^met^*	**Up**		*ns*	0.7947	***0.0247***
***FANCA***	*BRCA1^met^*	**Up**		*ns*	0.6261	***0.02817***
***MGMT***	*BRCA1^met^*	**Up**		*ns*	0.595	***0.005023***

LogFC BRCA1^mut^, log2 fold-change between BRCA1^mut^ and BRCA^wt^ cases; LogFC BRCA^met^, log2 fold-change between BRCA1^eut^ and BRCA^wt^ cases; up, significantly upregulated; down, significantly downregulated; ns, not significant (p-value > 0.05); EOC, epithelial ovarian cancer.

Bold indicates significant p-values.

Although all these DE genes are known to be involved in DNA damage and repair systems, such as HR, base excision repair (BER), nucleotide excision repair (NER), mismatch repair (MMR), non-homologous end joining (NHEJ), and chromatin remodeling (CR) (**Table 1**), we wondered if crosstalk between these systems would exist. **Supplementary Figure 1** shows that these DE genes are connected by shared function or are part of a physical complex. In addition, when considering the direct interactions only (**Supplementary Figure 1B**), STRING analysis identified a main group including 12 interacting proteins, whereas *HELLS-WDR76*, *MGMT*, *DDB2*, and *DTX3L-PARP9-PARP12* do not directly bind a *BRCA1*-associated complex.

### 3.3 BRCA1met EOCs show a distinctive gene expression profile

The comparative analysis between EOC groups was extended to all 750 genes. Remarkably, a total of 196 genes were differentially expressed in *BRCA1*-defective cases with respect to *BRCA^wt^* EOC (**Supplementary Table 3**). Interestingly, among all the pathways activated or repressed in *BRCA1*-defective EOCs compared to *BRCA^wt^* cases, both the *BRCA1^met^* and *BRCA1^mut^* groups shared significant upregulation of proliferation signature (p = 0.02232 and p = 0.01051, respectively), suggesting a more aggressive behavior of these EOCs in comparison to HR proficient ovarian cancers (**Table 2**). Regarding anti-tumor immune activity, no difference in Tumor Inflammation Signature (TIS) was observed among all classes of EOCs, suggesting a general “cold” immunological profile. Of note, 53 out of 196 genes resulted in DE in both *BRCA1^met^* and *BRCA1^mut^* EOCs in comparison to *BRCA^wt^* cases, possibly indicating a common pathogenetic profile driven by *BRCA1* deficiency. Twenty-seven genes were downregulated, while 26 genes were upregulated in both *BRCA1*-defective groups. The 27 downregulated transcripts were significantly enriched by genes involved in cell attachment to extracellular matrix (ECM), cell death, and apoptosis by regulating p53, WNT, RAS, or PI3K/AKT pathways, whereas the 26 upregulated transcripts were enriched by genes involved in cell cycle regulation, DNA damage and repair, chromosome organization, and immune response (**Supplementary Table 3**).

**Table 2:** Significantly upregulated/downregulated gene signature in *BRCA*-defective cases (*BRCA1^mut^* and *BRCA1^met^*) compared to *BRCA^wt^* EOCs.

Signature	EOC class	up/down	logFC *BRCA1^mut^*	p-Value *BRCA1^mut^*	logFC *BRCA1^met^*	p-Value *BRCA1^met^*
***Proliferation***	Both	**Up**	0.823	***0.02232***	0.7994	***0.01051***
***Mast Cells***	*BRCA1^met^*	**Down**		*ns*	−0.8518	***0.01336***
***TH1 Cells***	*BRCA1^met^*	**Down**		*ns*	−0.4948	***0.0405***
***IFN Gamma***	*BRCA1^met^*	**Up**		*ns*	1.256	***0.04116***
***NK CD56dim***	*BRCA1^met^*	**Down**		*ns*	−0.6014	***0.04427***
***MHC2***	*BRCA1^mut^*	**Up**	1.043	***0.0242***		*ns*
***IFN Downstream***	*BRCA1^mut^*	**Up**	0.55	***0.04362***		*ns*

LogFC BRCA1^mut^, log2 fold-change between BRCA1^mut^ and BRCA^wt^ cases; LogFC BRCA^met^, log2 fold-change between BRCA1^eut^ and BRCA^wt^ cases; up, significantly upregulated; down, significantly downregulated; ns, not significant (p-value > 0.05); EOC, epithelial ovarian cancer.

Bold indicates significant p-values.

**Figure 2:**
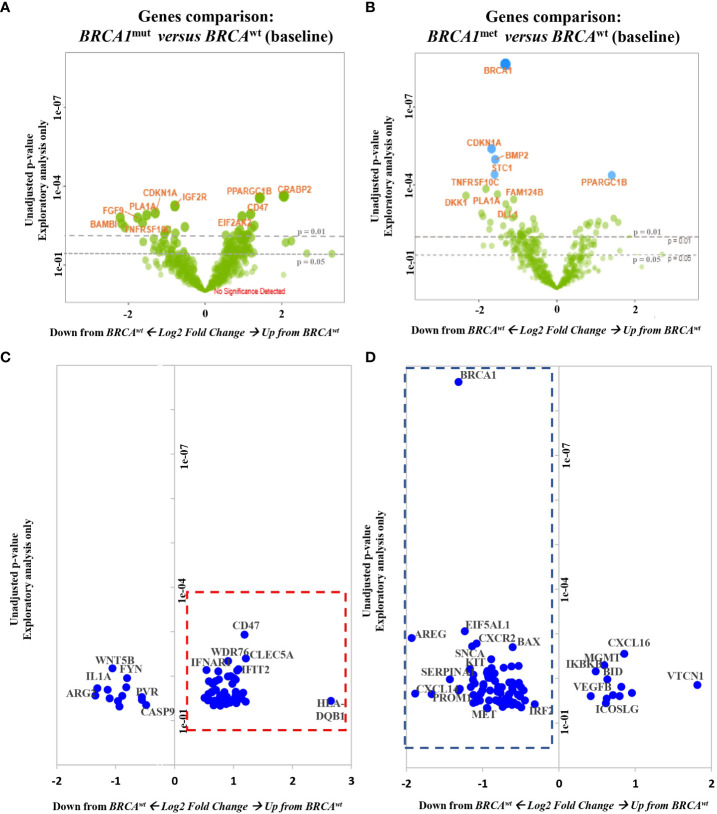
Top genes that are differentially expressed in *BRCA1^mut^* and *BRCA1^met^* EOCs. Volcano plots display each gene’s fold change (x-axis) and significance (p-value, y-axis) between **(A, C)** *BRCA1^mut^* (B group) and *BRCA^wt^* (A group) samples and **(B, D)** between *BRCA1^met^* (C group) and *BRCA^wt^* (A group) samples. Genes that have greater statistical significance will produce points that are both larger and darker in hue, in addition to appearing higher on the plot. Genes that have greater differential expression versus the baseline group (*BRCA* ^wt^, A group) appear further from the center of the plot. Genes further to the right indicate an increase in expression, and genes further to the left indicate a decrease in expression relative to the baseline group. Horizontal lines indicate 0.01 and 0.05 p-values. Blue dots indicate adjusted p-values lower than 0.05. Green dots indicate adjusted p-values higher than 0.05. The lower panels focus only on the 196 significantly DE genes in *BRCA1^mut^* and *BRCA1^met^* EOCs: red-dotted square indicates upregulated genes in *BRCA1^mut^*; blue-dotted square indicates downregulated genes in *BRCA1^met^*. EOC, epithelial ovarian cancer; DE, differentially expressed.

Interestingly, a total of 58 out of 196 genes were DE exclusively in *BRCA1^mut^* EOCs (**Supplementary Table 3**, **Figure 2A**): only 13 genes were downregulated, while 45 genes (78%) were significantly upregulated. In detail, the 13 downregulated transcripts were significantly enriched by genes involved in cell–cell adhesion and regulation of apoptotic process (such as *ARG2*, *SERPINB5*, *PVR*, *CASP9*, and *WNT5B* genes); instead, the 45 upregulated genes were significantly enriched by functions as innate immune response and cell cycle regulation (**Supplementary Table 3**, **Figure 2C**). In fact, both MHC2 and interferon (IFN) DOWNSTREAM signatures were significantly upregulated in this subset of EOCs (**Table 2**).

Finally, 85 out of 196 genes were DE exclusively in the *BRCA1^met^* group (**Supplementary Table 3**, **Figure 2B**): 73 of these genes (86%) were significantly downregulated, whereas only 12 genes were upregulated in comparison to *BRCA^wt^* EOCs. The 73 downregulated transcripts were enriched by genes involved in the regulation of cell death, cytokine–cytokine interaction, and development, while the 12 upregulated transcripts were enriched by genes of regulation of the immune system such as *ICOSLG*, *BID*, *CXCL16*, and *VTCN1* and DNA repair genes (**Supplementary Table 3**, **Figure 2D**). In detail, in *BRCA1^met^* EOCs, a significant downregulation of MAST CELLS, TH1 CELLS, and NK CD56DIM signatures were identified, whereas a significant upregulation of IFN GAMMA signature was observed (**Table 2**).

In spite of a cold immune signature background, both *BRCA1*-defective groups show a slightly activated immune response mediated by IFN gamma pathways.

Overall, comparing the expression profiles of the *BRCA1^mut^* and *BRCA1^met^* groups, *BRCA1^met^* EOCs show a significant strong gene downregulation with respect to *BRCA1^mut^* cases (chi-square p-value < 0.0001, **Figures 2C, D**), suggesting that, irrespective of the DE gene functions, remarkably different oncogenic mechanisms underlying the tumor initiation phase of *BRCA1^mut^* or *BRCA1^met^* EOC exist.

## 4 Discussion

Much clinical evidence demonstrated that *BRCA* pathogenetic variants are positive predictors of response to platinum-based and PARPi therapies (3, 11, 21–23). HRD status resulting from the disruption of genes involved in the HR pathway is currently considered a useful clinical tool to detect PARPi-eligible cases (5). However, HRD status embraces a class of heterogeneous tumors showing a wide spectrum of pathogenetic mechanisms, including somatic, germline, or epigenetic alteration of several HR genes. Thus, HRD status might not be a suitable marker to predict specific therapy responses and patient outcomes. Despite this, the HRD test is spreading through many laboratories, without a consolidated knowledge about the overlap between the HRD test and, in particular, *BRCA* methylation that characterized approximately 20% of BRCA wild-type EOCs (9). To date, no studies have focused on the transcriptional profiles of *BRCA* methylated cases, and, as a matter of fact, we still do not know if this subset of EOC is similar, and to what extent, to *BRCA* mutated cases.

In this study, we compared the expression profile of *BRCA1^met^* and *BRCA1^mut^* EOCs with respect to carefully selected EOC groups without germline or somatic defects in HR genes (namely, *BRCA^wt^* EOCs) using a highly sensitive and precise transcript quantification method (24).

As a preliminary step, we verified that *BRCA1^met^* cases had a downregulation of BRCA1 transcript, irrespective of the degree of promoter methylation. This is in line with what was reported in our previous work by using a real-time approach on a small subset of *BRCA1^met^* EOCs (8). Of note, the setting of a cutoff of methylation with a clinical meaning is a tricky issue, even in the light of the chromosome instability with chromosome 17 aneuploidies that characterize virtually all EOCs (3, 25, 26), the tumor purity of each analyzed sample, the type of method used for methylation quantification, and the size of validation cohorts (27, 28). On the contrary, in *BRCA1^mut^* cases, the gene dosage was not altered, neither in cases showing a truncating mutation, as it is expected that the loss of function is due to a pathogenetic non-functional protein variant. Indeed, epigenetic and genetic *BRCA1* alterations are mutually exclusive as extensively demonstrated in the literature (3, 9, 28, 29).

Compared to *BRCA^wt^* EOC, both *BRCA1^mut^* and *BRCA1^met^* cases display an upregulation of many genes related to DNA damage and repair system, among them *BRCA2* transcript, supporting the concept, better explained elsewhere (30), that the perturbation of a particular gene’s function may alter the expression of other genes within the same network as a mechanism to try to maintain cellular wellness (31, 32). Moreover, both subsets show the upregulation of pathways related to cell proliferation and interferon gamma. These findings were extensively demonstrated for *BRCA1^mut^* cases; in fact, several authors proved that BRCA1 loss leads to transcriptional reprogramming in tumor cells involving type I IFN signaling (33–35).

Considering all the 196 DE transcripts, the analysis of single genes highlighted a prevalent “upregulation profile” in *BRCA1^mut^* and a prevalent “downregulation profile” in *BRCA1^met^* (**Figure 2**). This finding suggests that the pathogenetic mechanisms of *BRCA1^mut^* and *BRCA1^met^* may be very different and that widespread methylation may characterize the initial tumorigenesis of the latter group. Interestingly, many authors have reported that a subset of ovarian cancers can be characterized by extensive gene hypermethylation, suggesting the existence of a CpG Island Methylator Phenotype (CIMP) in the ovarian site (36–39). This phenotype has been deeply characterized and illustrated in colorectal cancer (CRC), where CIMP-CRC has been classified as a distinctive entity, with *MLH1* methylation as the flag of a diffuse methylator pattern (40). Transposing this concept to the ovarian site, *BRCA1* methylation could be the hint to identify such cases. Moreover, the presence of widespread methylation might prompt further investigation to identify epigenetic drugs, as well as to address potential different clinical outcomes as reported for other tumor sites (41).

Curiously, despite that the genes deregulated in the *BRCA1^mut^* and *BRCA1^met^* EOCs are remarkably different, the enrichment analysis suggests that, overall, the two groups share the same perturbed pathways. This finding, derived from the study of a limited proportion of the transcriptome, i.e., 750 genes grouped in 48 pathways, deserves to be further investigated by global expression analysis. In fact, *BRCA1^mut^* and *BRCA1^met^* EOCs may result as different entities, presenting even more remarkable differences than those highlighted by this study.

Furthermore, these findings indicate the potential expansion of this approach to other tumor sites where BRCA is implicated, aiming to explore whether methylation is a prevalent characteristic across different types of tumors.

In summary, this study represents the first instance where a correlation has been established between promoter methylation, as identified through a BRCA1 methylation test developed in-house, and transcript downregulation. Moreover, *BRCA1^met^* EOCs are characterized by diffuse gene downregulation, suggesting that this subset shows a pathogenetic mechanism remarkably different with respect to *BRCA1^mut^* cases. Even if the expression profile of many genes related to DNA damage and repair system is shared between *BRCA1^mut^* and *BRCA1^met^* EOCs, supporting that *BRCA1^met^* EOCs may benefit from PARPi therapies, our data demonstrate that *BRCA1^mut^* and *BRCA1^met^* EOCs have different expression profiles underlying different mechanisms of carcinogenesis that can influence the type of responses to therapies and disease recovery.

## Data availability statement

The data presented in this study are available in the article or in supplementary material.

## Ethics statement

The studies involving humans were approved by Research Ethics Committee of ATS Insubria (ID 238/2018). The studies were conducted in accordance with the local legislation and institutional requirements. The participants provided their written informed consent to participate in this study.

## Author contributions

NS: Data curation, Writing – original draft. LL: Data curation, Formal Analysis, Writing – original draft. SF: Data curation, Formal Analysis, Writing – original draft. IC: Data curation, Writing – review & editing. SR: Data curation, Writing – review & editing. CA: Data curation, Writing – original draft. AC: Data curation, Writing – review & editing. JC: Data curation, Writing – review & editing. FS: Conceptualization, Funding acquisition, Writing – review & editing. MT: Conceptualization, Funding acquisition, Supervision, Writing – review & editing.

## Supplementary material

Supplementary Figure 1 Protein–protein networks of significantly down/up-regulated genes in BRCA-defective EOCs by using STRING database. Network nodes represent proteins: splice isoforms or post-translational modifications are collapsed, i.e. each node represents all the proteins produced by a single, protein-coding gene locus. **(A)** Edges represent protein-protein associations: associations are meant to be specific and meaningful, i.e. proteins jointly contribute to a shared function; this does not necessarily mean they are physically binding to each other. **(B)** Edges represent shared physical complex: the edges indicate that the directly linked proteins are part of the same physical complex; commonly in large complexes this may not signify they are directly binding to each other*. Legend: black asterisks indicate differential expressed (DE) genes for both BRCA1^mut^ and BRCA1^met^ EOCs; orange asterisks indicate DE genes in only BRCA1^mut^ EOCs; green asterisks indicate DE genes in only BRCA^met^ EOCs. Edges thickness indicates the level of confidence of the interaction: lighter line indicates high confidence rate (STRING score 0.700); darker line indicates highest confidence rate (STRING score 0.900).*
