# Supplementary figures and images for: Similarities and differences in gene expression profiles of BRCA1 methylated and mutated epithelial ovarian cancers
Source: Front Oncol. 2023 Oct 3;13:1268127. doi: 10.3389/fonc.2023.1268127 (PMC10579792; doi:10.3389/fonc.2023.1268127)

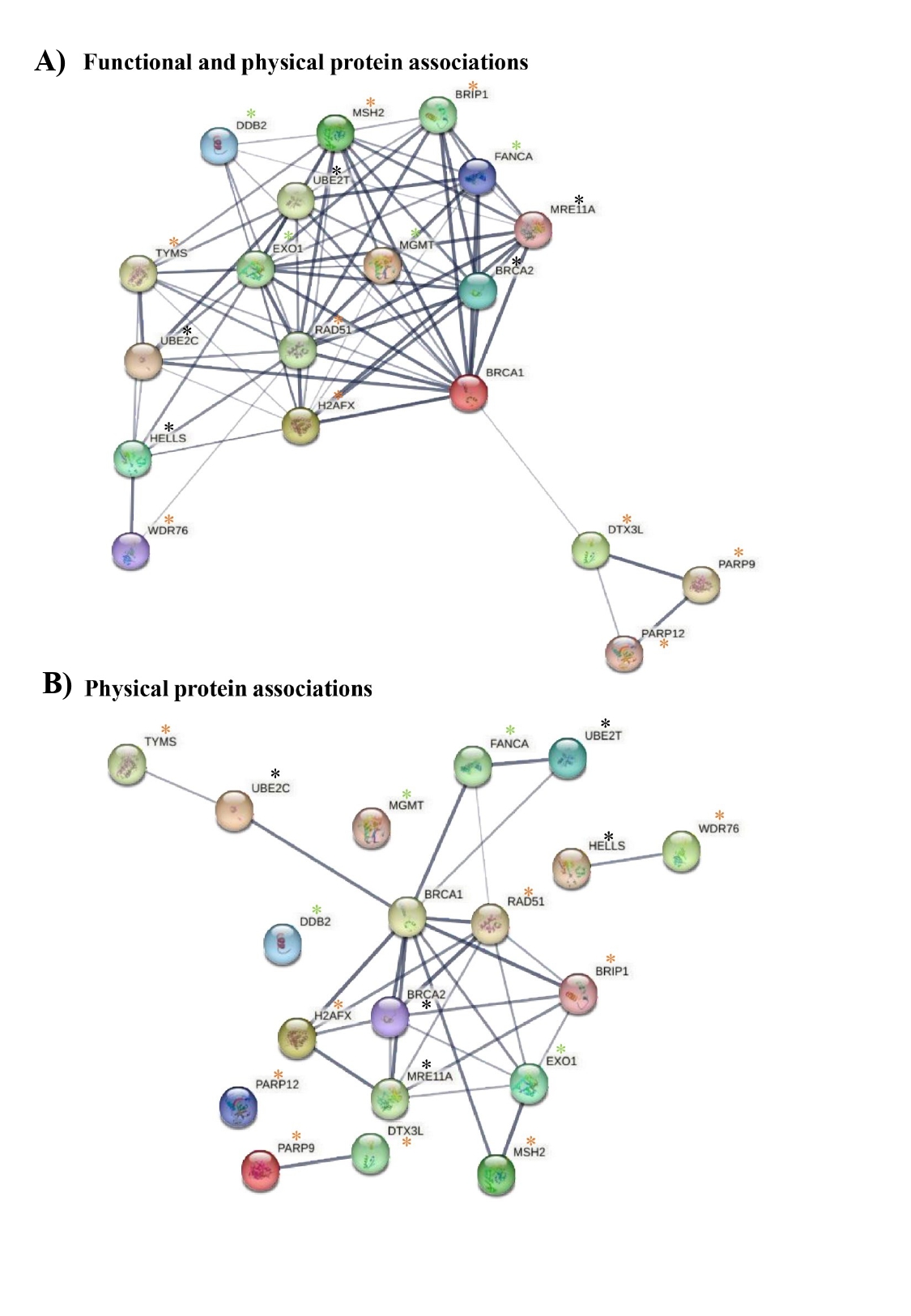

Supplement: Supplementary Figure 1 — Protein–protein networks of significantly down/up-regulated genes in BRCA-defective EOCs by using STRING database. Network nodes represent proteins: splice isoforms or post-translational modifications are collapsed, i.e. each node represents all the proteins produced by a single, protein-coding gene locus. (A) Edges represent protein-protein associations: associations are meant to be specific and meaningful, i.e. proteins jointly contribute to a shared function; this does not necessarily mean they are physically binding to each other. (B) Edges represent shared physical complex: the edges indicate that the directly linked proteins are part of the same physical complex; commonly in large complexes this may not signify they are directly binding to each other. Legend: black asterisks indicate differential expressed (DE) genes for both BRCA1mut and BRCA1met EOCs; orange asterisks indicate DE genes in only BRCA1mut EOCs; green asterisks indicate DE genes in only BRCAmet EOCs. Edges thickness indicates the level of confidence of the interaction: lighter line indicates high confidence rate (STRING score 0.700); darker line indicates highest confidence rate (STRING score 0.900). [file Image_1.jpg]
